# Trends in 4th−12th grade students' aerobic capacity and muscular strength and endurance: New York City public school students, 2006–2019

**DOI:** 10.3389/fpubh.2026.1682913

**Published:** 2026-02-18

**Authors:** Sophia Day, Caroline Nguyen, Kira Argenio, Kevin Konty, Sydney S. Dougan, Emily D'Agostino, Cody D. Neshteruk, Brooke E. Wagner, Hannah R. Thompson

**Affiliations:** 1 Office of School Health, Research and Analytics, New York City Department of Health and Mental Hygiene, New York, NY, United States; 2 Department of Community Health Science, School of Public Health, University of California, Berkeley, Berkeley, CA, United States; 3 Department of Population Health Sciences and Department of Orthopaedic Surgery, Duke University School of Medicine, Durham, NC, United States

**Keywords:** cardiorespiratory fitness (CRF), elementary students, muscular endurance, muscular strength, secondary students

## Abstract

**Background:**

Systematically monitoring youth fitness over time is critical for designing and implementing policies and interventions to maintain/increase youth fitness and reduce the burden of non-communicable disease. This study describes trends in student fitness over school years 2006/07–2018/19, the longest period for which US-based data are available.

**Methods:**

This observational, longitudinal study uses 13 years of population-weighted data from the New York City (NYC) FITNESSGRAM from 2,272,575 unique students (8,523,877 student-year observations) in 4th−12th grades from 1,721 public schools. Sex-and age-specific performance was assessed using healthy fitness zones (HFZ), validated performance standards indicating whether individual fitness was sufficient for good overall health. Adjusted generalized estimating equations determined the prevalence of being in the HFZ for aerobic capacity, push-ups, and curl-ups for all students, and by student sex, grade level, race/ethnicity, and home neighborhood socioeconomic status (SES).

**Results:**

In 2006/07, 24.7% of students were in the HFZ for aerobic capacity, 55.3% for push-ups, and 65.0% for curl-ups. By 2018–19, 35.6% were in the HFZ for aerobic capacity (a relative increase of 44.1% from baseline; *p* < 0.001); 56.1% (SD = 0.56) for push-ups (1.4% relative increase; *p* < 0.001); and 70.5% (SD = 0.54) for curl-ups (8.5% relative increase; *p* < 0.001). Stratified models demonstrated persistent differences in the proportion of students in the HFZ by sex, school level, and home neighborhood SES. Gaps between non-Hispanic White compared to non-Hispanic Black and Hispanic students grew slightly.

**Conclusion:**

Thirteen years of data from the largest school district in the nation demonstrate overall improvements in student fitness performance on all three tests, with the largest gains seen for aerobic capacity. However, continued work is necessary to address persistent disparities in NYC youth aerobic capacity and muscular strength and endurance by sex, grade level, race/ethnicity, and socioeconomic status.

## Introduction

Adequate physical fitness in youth is both an indicator of present-day health and a predictor of health in later life, reducing the risk for cardiovascular disease, type 2 diabetes, metabolic syndrome, breast and colon cancer, and dementia and Alzheimer's disease (1–5). Furthermore, higher youth cardiorespiratory fitness is associated with improved health outcomes (i.e., lower risk for metabolic syndrome later in life) and academic performance (4, 6, 7). Appropriate muscular strength (muscular force generation) and endurance (repeated muscle use over time without tiring) are linked with positive body composition outcomes, as well as better cardiorespiratory and metabolic health, though the evidence is notably less robust (7).

Despite the known importance of fitness, only 42% of 12–15 year-olds in the United States (US) had adequate cardiorespiratory fitness in 2012, the most recent year in which such national data are available (8). This, combined with other fitness indicators (muscular strength and muscular endurance), led the 2022 US Report Card on Physical Activity for Children and Youth to give American youth a C- for physical fitness (9). Furthermore, disparities in youth fitness across race, ethnicity, poverty, and sex endure—with White, high income, and male students consistently performing better than their non-White, low income, and female counterparts—which predicts persistent inequities in chronic disease into adulthood (9–12).

Given the critical impact of physical fitness on child and adolescent health, it is necessary to systematically monitor and assess changes over time, in order to design and implement policies and interventions aimed to maintain or increase youth physical fitness and reduce the burden of non-communicable disease (13, 14). However, population-based studies on physical fitness trends in youth are challenging to administer given their high cost and burden on participants (13). As a result, reliable population-level data on the current status and secular trends in youth physical fitness are rarely reported on at a national level. In fact, the last available national data is from more than a decade ago (8). At both the national and state levels, there are no known systems in place to longitudinally monitor youth fitness. School-based surveillance systems, therefore, can play an important role in monitoring youth fitness over time.

The FITNESSGRAM, a multi-component physical fitness assessment, is the most widely-used tool to assess and report on school-aged youths' fitness across the US and has been used to test more than 22 million students in schools across all 50 states (15). The FITNESSGRAM objectively measures health-related components of youth fitness, including cardiorespiratory fitness and muscular strength and endurance (16).

While prior research using FITNESSGRAM data has examined student fitness overall (using a combined count of the number of age- and sex-specific standards met) (17), there is limited longitudinal data describing the prevalence of achieving age- and sex-specific standards for individual tests, precluding our ability to examine trends in individual fitness components over time. Given the strong associations between aerobic capacity, current and future health, and academic outcomes, understanding differences in student fitness performance disaggregated for aerobic capacity and muscular strength and endurance is critical to helping identify the most relevant interventions to address inequities.

The New York City (NYC) Department of Education (DOE), the largest school district in the nation and among the most racially/ethnically and socioeconomically diverse, in partnership with the NYC Department of Health and Mental Hygiene, has created one of the largest school-based student fitness surveillance system in the country. Since school year 2006/07, they have combined data from multiple sources (DOE administrative databases, the American Community Survey, the US Census, and NYC FITNESSGRAM assessments) into a single comprehensive record for each student for each year of attendance in public school. This longitudinal, student-level analytic dataset is used to define the NYC public school student population and can be used to construct prevalence and trend estimates for fitness outcomes. Given the number of students included (approximately 850,000 annually) and the racial/ethnic and socioeconomic diversity of the students represented in this dataset, findings from studies utilizing this sample are highly policy- and practice-relevant.

The objective of this study is to describe longitudinal trends in the proportion of 4th−12th grade New York City public school students meeting age- and sex-specific standards for individual tests of aerobic capacity and muscular strength and endurance over 13 school years (2006/07–2018/19), the longest period for which these data have been available. Capitalizing on individually measured fitness performance data, we present trends for all students, as well as by key school and sociodemographic characteristics associated with youth fitness: sex, grade level, race/ethnicity, and socioeconomic status. Results from this study can help identify policy and programmatic interventions designed to better address persistent inequities in youth fitness outcomes.

## Materials and methods

### Data source and study population

Data for this 13-year longitudinal study were taken from the NYC FITNESSGRAM dataset, which is managed by both the NYCDOE and the NYC Department of Health and Mental Hygiene (DOHMH), for school years 2006/07 (first year available) through 2018/19 (most recent complete year available). NYC FITNESSGRAM includes student fitness assessments collected annually by NYCDOE for approximately 860,000 public school students per year. This dataset included annual fitness assessment data collected by NYCDOE for 2,736,061 NYC public school students (14,650,964 student-year observations) from kindergarten through 12th grade from 2,297 schools (18). Inclusion criteria for the current study were: (1) enrollment in traditional education districts [excluding three districts which educate special education, charter, and adult students and are not required to administer the FITNESSGRAM, which excluded 416,035 students (1,323,867 student-year observations)]; (2) aged 9–19 years as of December 31st of the school year and enrolled in 4th−12th grades (grades in which the aerobic capacity and muscular strength and endurance tests were conducted), which excluded 2,608,925 students (4,695,279 student-year observations); and (3) having valid aerobic capacity, push-up, and curl-up data within the given school year, which excluded 956,030 students (1,883,829 student-year observations). The final unweighted dataset included 1,631,523 unique students (6,748,265 student-year observations). Data for this analysis were deidentified, accessed on September 12, 2023, and authors did not have any information that could identify individual students. Due to the anonymous nature of the data, this study was not considered Human Subjects Research and did not require Institutional Review Board approval.

### FITNESSGRAM measures

The NYC FITNESSGRAM is administered from September to May each school year by formally trained physical education teachers using standardized equipment. The NYCDOE mandates schools have at least 85% of eligible students complete the assessment battery each school year, which consists of five assessments designed to measure distinct components of health-related fitness (15). Aerobic capacity is evaluated using estimates of VO_2_max (the maximum rate at which the cardiovascular, respiratory, and muscular systems take in, transport, and use oxygen during physical activity), assessed by the Progressive Aerobic Cardiovascular Endurance Run (PACER Test). During the PACER, students complete as many shuttle runs back and forth across a 15-meter course as possible, in time to an audio recording that is paced to get faster every minute. Muscular strength and muscular endurance are measured via curl-ups (conducted with flexed knees and feet unanchored) and push-ups (performed at a 90° elbow angle). Both the curl-up and push-up tests are performed at a specified pace until form is broken or the pace is not met.

The NYC FITNESSGRAM assessments of body composition [assessed using body mass index (BMI)], and musculoskeletal flexibility (measured by the sit-and-reach), are not included in the present study. While body composition is associated with fitness in youth, it cannot be characterized as a fitness test, and has thus been excluded (19, 20). Flexibility has also been omitted, due to the lack of consistent evidence on its association with youth fitness (21), as well as recent questions on the validity of this assessment as a measure of flexibility (22).

This study reports on PACER, curl-up, and push-up test data, which have been deemed reliable and valid measures of cardiorespiratory fitness and abdominal and upper body strength and endurance, respectively (15). Further, these three fitness tests have been shown to predict cardiovascular disease risk, and are the foundation for recommendations for health monitoring systems to include youth fitness testing (15). The primary outcomes were three binary (yes/no) variables indicating whether the student met the performance criteria for the Cooper Institute's sex- and age-specific Healthy Fitness Zones (HFZ) for each of the three tests (PACER, curl-up, and push-up). The HFZs were chosen over continuous measures because the HFZs indicate whether a student met criterion-referenced sex- and age-specific physical fitness levels on each test and provide a standardized indication of present and future health (15, 23). HFZ criteria were standardized for all PACER, push-up, and curl-up tests and years of testing (2006/07–2018/19) based on the Cooper Institute's most current HFZ criteria (15).

### Covariates

Available data on established correlates of youth physical activity were included as covariates (24, 25). Fixed (due to data processing procedures) covariates included: student sex (male/female); place of birth (US or foreign-born); primary language spoken at home (English, Spanish, or other); and race/ethnicity (Asian/Pacific Islander, non-Hispanic Black, Hispanic/Latino, non-Hispanic White, other/multiple races). Students not identifying a specific race/ethnicity (due to parent refusal or missing data) or reporting multiple races were classified as “other.” American Indian/Native Alaskan students, a distinct racial classification, are also grouped as “other” due to the small sample size. Although the classification of the “other” race/ethnicity category is not coherent, we use it for modeling and analysis with the aim to reduce its use. In consideration of these limitations, prevalence estimates for students classified as “other” race/ethnicity are not provided.

Time-varying covariates (which could change annually) included age, grade type [elementary (grades 4th−5th); middle (6th−8th); and high school (9th−12th)], and student socioeconomic status (SES). Student eligibility for free or reduced-price meals (FRPM) through the National School Lunch Program (often used as a proxy for SES), is presented cross-sectionally, as it is an oft-used marker of SES in such school-based studies. However, it is not included in the longitudinal analysis, as FRPM data were not collected consistently across study years due to changes in eligibility criteria. Instead, longitudinal models include student home neighborhood poverty level as a proxy for SES, due to its measurement stability over time. Student home neighborhood SES was determined for students' home census tracts using the American Community Survey 2008–2012 poverty data by census tract (defined according to 2010 Census boundaries) and categorized as very low (0 to < 10% of individuals living below 100% of the Federal Poverty Level), low (10 to < 20%), high (20 to < 30%), and very high (30%−100%). Missing covariate values were resolved, when possible, using information for the same student from the closest year available.

### Statistical analysis

Observations with complete date of birth, sex, PACER, push-up, and curl-up data were weighted to be representative of the NYC public school 4th−12th grade enrollment population for each school year (2006/07–2018/19), accounting for individual- and school-level characteristics. The procedure is similar to post-stratification for non-random survey nonresponse (26, 27). In short, for each school year and grade type (elementary, middle, high), observations with complete data were weighted using standard raking procedures to match marginal control totals for: age, race by neighborhood, grade by sex by neighborhood, and individual student household poverty by neighborhood (18). Descriptive statistics were computed to summarize sample characteristics for both the unweighted and weighted populations (unweighted data are available in the Appendix) for each school year. All other analyses were performed using the weighted dataset.

To determine the prevalence of being in the HFZ for PACER, curl-up, and push-up for each school year (2006/07–2018/19; cross-sectional models) for all students, generalized estimating equations adjusted for student sex, age, race/ethnicity, place of birth, primary language spoken at home, and home neighborhood poverty level, with a random effect for school, were used. To examine longitudinal trends in population-level fitness, a multivariate model for each of the three outcomes (aerobic capacity, push-ups, curl ups) was built where the probability of being in the HFZ was modeled on time (an integer value that increases from 0 to 12 corresponding to the 2006/07–2018/19 school years). Standard errors were clustered at the school- and student-level, and models were adjusted for student age, sex, race/ethnicity, place of birth, primary language spoken at home, and neighborhood SES.

To examine differences by key subgroups of interest, we added an interaction term between time and demographic variables (student sex, grade level, race/ethnicity, and home neighborhood SES) to each model to assess relative differences in trends across subgroups. If interaction was present (i.e., for all statistically significant effect modification models), adjusted logistic mixed effects models were then run stratified by student sex, grade level, race/ethnicity, and home neighborhood SES level to illustrate annual subgroup differences. Analyses were conducted in SAS v9.4 (SAS Institute Inc., Cary, NC, United States) and figures were created in RStudio (v4.3.1) (Posit Software, PBC, Boston, MA, United States).

## Results

The weighted analytic sample included 2,272,575 unique students (8,523,877 student-year observations) from 1,721 NYCDOE traditional public schools. In the final study year (2018/19), the weighted sample included 636,983 students, of which 48.9% were female (Table 1; unweighted data are available in Supplementary Appendix Table 1). Nearly half (45.1%) of students were in high school (grades 9–12), 32.5% in middle school (grades 6–8), and 22.4% in elementary school (grades 4–5). Forty-one percent of students were Hispanic, 23.3% non-Hispanic Black, 18.2% Asian and/or Pacific Islander, and 15.4% non-Hispanic White. Most students (79.2%) were born in the US, spoke English at home (53.9%) and qualified for free or reduced-price meals (74.2%, a marker of household poverty).

**Table 1:** Sociodemographic characteristics of weighted New York City public school student sample (*n*_weighted_ = 2,272,575 students, *n*_weighted_ = 8,523,877 observations), grades 4–12.

**Characteristics**	**2006/07**	**2007/08**	**2008/09**	**2009/10**	**2010/11**	**2011/12**	**2012/13**	**2013/14**	**2014/15**	**2015/16**	**2016/17**	**2017/18**	**2018/19**
All students, % (*n*)	100 (673,392)	100 (672,245)	100 (666,369)	100 (670,522)	100 (668,508)	100 (662,793)	100 (654,392)	100 (648,624)	100 (643,965)	100 (642,784)	100 (639,938)	100 (643,362)	100 (636,983)
**Sex**													
Female	49.4	49.5	49.4	49.3	49.2	49.2	49.1	49.0	48.9	48.9	48.9	48.9	48.9
Male	50.6	50.5	50.6	50.7	50.8	50.8	50.9	51.0	51.1	51.1	51.1	51.1	51.1
**Grade level**													
Elementary (4th−5th grades)	21.6	21.1	21.1	21.3	21.5	21.5	21.7	21.8	22.2	22.5	23.0	22.7	22.4
Middle (6th−8th grades)	33.3	32.5	32.4	32.2	32.1	32.2	32.3	32.4	32.0	31.6	32.0	32.0	32.5
High (9th−12th grades)	45.1	46.3	46.4	46.5	46.4	46.2	46.0	45.8	45.8	45.6	45.0	45.3	45.1
**Race**													
Asian and/or Pacific Islander	13.9	14.3	14.7	15.4	15.8	16.1	16.5	16.9	17.1	17.5	18.0	18.1	18.2
Non-Hispanic Black	32.9	32.6	32.0	31.1	30.3	29.5	28.6	27.5	26.6	25.7	24.7	24.0	23.3
Hispanic	39.0	39.4	39.6	39.6	39.9	39.9	40.0	40.2	40.5	40.5	40.5	40.8	41.0
Non-Hispanic White	13.4	13.2	13.2	13.3	13.5	13.8	14.2	14.4	14.6	14.9	15.2	15.3	15.4
Other^a^	0.7	0.5	0.5	0.5	0.6	0.6	0.8	1.0	1.2	1.4	1.6	1.8	2.0
**Primary language spoken at home**													
English	55.5	54.5	55.7	55.7	55.5	55.3	55.2	54.9	54.8	54.7	54.1	53.9	53.9
Spanish	27.3	27.9	26.8	26.3	26.1	25.9	25.7	25.6	25.6	25.4	25.3	25.3	25.3
Other language	17.2	17.6	17.5	18.0	18.4	18.8	19.1	19.4	19.6	19.9	20.5	20.7	20.8
**Place of birth**													
US	78.0	77.5	78.5	78.7	78.5	78.9	79.3	79.5	79.7	79.9	79.5	79.2	79.2
Foreign	22.0	22.5	21.5	21.3	21.5	21.1	20.7	20.5	20.3	20.1	20.5	20.8	20.8
**Household poverty** ^b^													
Low	22.7	23.9	26.2	16.5	16.3	18.3	26.5	25.8	26.9	28.3	28.4	24.3	25.8
High	77.3	76.1	73.8	83.5	83.7	81.7	73.5	74.2	73.1	71.7	71.6	75.7	74.2
**Home neighborhood SES** ^c^													
Very-wealthy (0 to < 10%)	19.5	18.9	18.9	19.0	19.0	19.2	19.4	19.7	20.0	20.3	20.7	20.8	21.2
Wealthy (10 to < 20%)	28.1	27.5	27.1	27.2	27.3	27.4	27.4	27.5	27.6	27.7	28.0	28.1	28.3
Poor (20 to < 30%)	24.7	24.8	24.5	24.5	24.6	24.7	24.5	24.4	24.3	24.2	24.2	24.0	23.9
Very-poor (30%−100%)	27.8	28.9	29.5	29.3	29.0	28.7	28.7	28.3	28.1	27.8	27.1	27.1	26.6

^a^Students not reporting Hispanic, non-Hispanic Black, non-Hispanic White, or Asian/Pacific Islander race/ethnicity in a school year are classified as “other,” which includes those reporting multiple races, parent refusal, or missing data. While a distinct racial classification, American Indian/Native Alaskan students are also grouped as “other” due to the small sample size.

^b^Individual student household poverty (high vs. low) was based on student eligibility/non-eligibility for free/reduced price school meals through the National School Lunch Program which provides meal assistance according to household income at or below 185% of the federal poverty level.

^c^Neighborhood socioeconomic status was defined according to American Community Survey 2008–2012 data as the percentage of households in the students' home census tract living below the federal poverty threshold and defined according to the Census 2010 boundaries.

Table 2 presents the annual adjusted proportion of students in the HFZ for aerobic capacity, push-ups, and curl-ups across study years for all students combined (*n* = 2,272,575). Fitness test results improved for all three tests across the 13-year study period, with the greatest improvements seen in aerobic capacity. In 2006/07 (the first year data were available), 24.7% (SD = 0.97) of students were in the HFZ for aerobic capacity, 55.3% (SD = 1.20) were in the HFZ for push-ups, and 65.0% (SD = 1.50) were in the HFZ for curl-ups. By 2018–19, 35.6% (SD = 0.60) of students were in the HFZ for aerobic capacity (a relative increase of 44.1% from baseline; *p*-value for trend < 0.001); 56.1% (SD = 0.56) were in the HFZ for push-ups (a relative 1.4% increase; *p*-value for trend < 0.001); and 70.5% (SD = 0.54) were in the HFZ for curl-ups (a 8.5% relative increase; *p*-value for trend < 0.001).

**Table 2:** Adjusted prevalence^a^ of meeting Healthy Fitness Zone standards^b^ for cardiorespiratory fitness and muscular strength and endurance for New York City public school students grades 4–12 (*n*_weighted_ = 2,272,575 students), 2006/7–2018/19.

**Test**	**2006/07% ±SD**	**2007/08% ±SD**	**2008/09% ±SD**	**2009/10% ±SD**	**2010/11% ±SD**	**2011/12% ±SD**	**2012/13% ±SD**	**2013/14% ±SD**	**2014/15% ±SD**	**2015/16% ±SD**	**2016/17% ±SD**	**2017/18% ±SD**	**2018/19% ±SD**	**Relative change 2006/07–2018/19**	***p*-value for test for trend^c^**
Aerobic capacity	24.7 ± 0.97	23.7 ± 0.78	25.3 ± 0.69	26.7 ± 0.69	27.4 ± 0.68	28.7 ± 0.69	29.6 ± 0.68	31.2 ± 0.62	32.2 ± 0.61	34.2 ± 0.63	34.9 ± 0.61	35.7 ± 0.60	35.6 ± 0.60	44.1%	<0.001
Push-ups	55.3 ± 1.20	56.2 ± 0.67	55.8 ± 0.60	55.8 ± 0.63	56.0 ± 0.59	57.0 ± 0.59	57.6 ± 0.57	58.1 ± 0.58	59.1 ± 0.56	59.5 ± 0.57	58.8 ± 0.55	57.2 ± 0.55	56.1 ± 0.56	1.4%	<0.001
Curl-ups	65.0 ± 1.50	65.5 ± 0.87	65.5 ± 0.74	66.6 ± 0.71	68.1 ± 0.66	69.0 ± 0.62	69.8 ± 0.61	71.1 ± 0.58	72.3 ± 0.55	72.2 ± 0.55	71.4 ± 0.55	70.9 ± 0.54	70.5 ± 0.54	8.5%	<0.001

^a^Estimated school year proportions derived from generalized estimating equation logistic models adjusted for student sex, age, race/ethnicity, place of birth, primary language spoken at home, and home neighborhood poverty level, with a random effect for school.

^b^Based on whether the student met the performance criteria for the Cooper Institute's most recent sex- and age-specific Healthy Fitness Zones for each test.

^c^P-values for tests for trends over school years derived from logistic mixed effects models with a linear term for trend, adjusted for sex, age, race/ethnicity, place of birth, primary language spoken at home, and home neighborhood poverty level with random effects for student and school.

**Figure 1:**
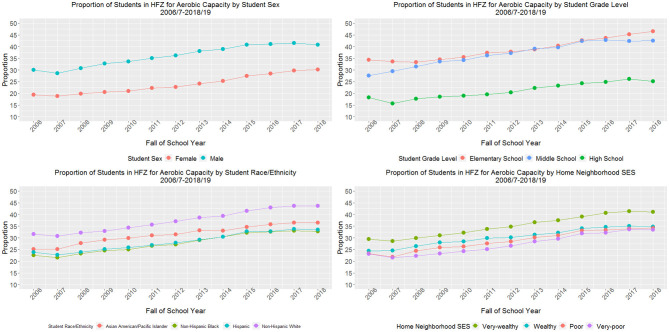
Proportion of students meeting cardiorespiratory fitness sex- and age-specific standards for New York City public school students grades 4–12 (*n*_weighted_ = 8,523,877 observations), 2006/7–2018/19.

For aerobic capacity (Figure 1), female students demonstrated a larger relative change from baseline compared with male students (60.6 vs. 42.7% in the HFZ). Yet, in 2018–19 only 30.2% of female students were in the HFZ compared to 40.8% of males (difference of 10.6 percentage points). Middle school students demonstrated a larger relative change in proportion of students in the HFZ (54.0%) compared with elementary (35.6%) and high school students (37.7%). However, in 2018–19 only 25.2% of high school students were in the HFZ compared with 46.5% of elementary and 42.5% of middle school students. Students across racial/ethnic groups demonstrated similar relative increases in the proportion of students in the HFZ (range of 38%−46%), but by the final study year, a lower proportion of Asian/PI (36.8%), non-Hispanic Black (32.8%), and Hispanic (33.4%) students were in the HFZ compared with non-Hispanic White students (43.7%). Similarly, students across the four strata of home neighborhood SES demonstrated similar relative increases in the proportion of students in the HFZ (range of 39–47%). However, by 2018–19, a lower proportion of students with very poor (33.4%), poor (34.2%), and wealthy (34.7%) home neighborhood SES were in the HFZ for aerobic capacity compared with students with very wealthy (41.1%) home neighborhood SES. For all stratified aerobic capacity models, the *p*-values for test for trend and the *p*-values for relative difference in trends by sex, grade level, race/ethnicity, and home neighborhood SES were < 0.001. These data are also available in tabular format in Supplementary Appendix Tables 2–5.

**Figure 2:**
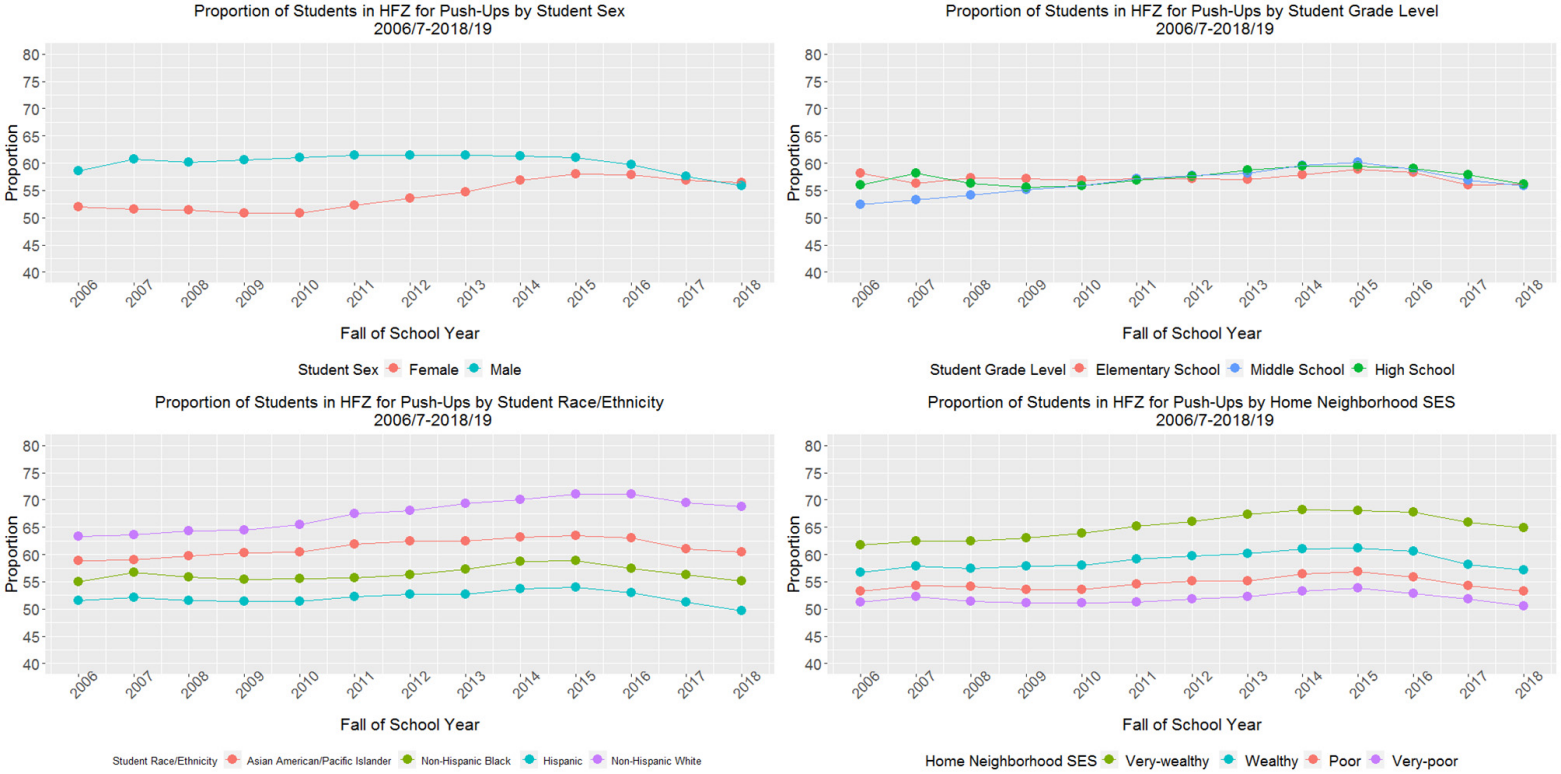
Proportion of students meeting sex- and age-specific muscular strength and endurance (push-up) standards for New York City public school students grades 4–12 (*n*_weighted_ = 8,523,877 observations), 2006/7–2018/19.

For push-ups (Figure 2), female students demonstrated a 9.5% relative increase in the proportion in the HFZ from baseline compared with an 8.2% decrease for male students, resulting in similar proportions of female (56.4%) and male (55.8%) students in the HFZ in 2018–19. While middle school students demonstrated a 6.7% relative increase in the proportion of students in the HFZ from baseline, high school students barely increased (0.4%) and elementary students declined (−3.6%), resulting in similar proportions of elementary (56.1%), middle (55.9%), and high (56.2%) school students in the HFZ in the final study year. Non-Hispanic White and Asian/PI students demonstrated relative increases in the proportion of students in the HFZ from baseline (8.7 and 2.7%, respectively), while non-Hispanic Black students saw very small change (0.2%) and Hispanic students saw a 3.5% decline from baseline. By the final study year, a lower proportion of Hispanic (49.7%) and non-Hispanic Black (55.1%) students were in the HFZ compared with Asian/PI (60.5%) and non-Hispanic White students (68.8%). Finally, students with very wealthy and wealthy home neighborhood SES demonstrated increases in the proportion of students in the HFZ (5.0 and 0.9% increase, respectively), whereas students with poor and very poor neighborhood SES showed decreases (−0.2 and −1.2%, respectively). Across study years, there was a clear dose-response relationship between home neighborhood SES and the proportion of students in the HFZ; by 2018–19, 50.6% of students with very poor, 53.2% with poor, 57.2% with wealthy, and 64.9% with very wealthy home neighborhood SES were in the HFZ for push-ups. For all stratified push-up models, the *p*-values for test for trend and the *p*-values for relative difference in trends by sex, grade level, race/ethnicity, and home neighborhood SES were < 0.001.

**Figure 3:**
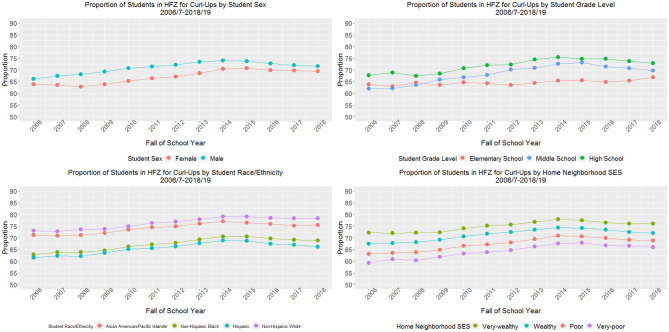
Proportion of students meeting sex- and age-specific muscular strength and endurance (curl-up) standards for New York City public school students grades 4–12 (*n*_weighted_ = 8,523,877 observations), 2006/7–2018/19.

For curl-ups (Figure 3), female students demonstrated a slightly greater relative change from baseline compared with male students (9.3 vs. 6.1% in the HFZ). In 2018–19 a similar proportion of female and male students were in the HFZ (69.4 and 71.6%, respectively). Middle school students demonstrated a larger relative change in proportion of students in the HFZ (12.4%) compared with elementary (4.5%) and high school students (7.7%). In 2018–19 66.8% of elementary, 69.7% of middle, and 72.9% of high school students were in the HFZ. Students across racial/ethnic groups demonstrated similar relative increases in the proportion of students in the HFZ (range of 5%−10%), but by the final study year, a lower proportion of Hispanic (66.2%) and non-Hispanic Black (68.9%) students were in the HFZ compared with Asian/PI (75.4%) and non-Hispanic White students (78.3%). Students with very poor home neighborhood SES saw the greatest relative increase from baseline (11.3%) compared with students with poor (9.2%), wealthy (6.7%), and very wealthy (5.5%) home neighborhood SES. Across study years, there was a clear dose-response relationship between home neighborhood SES and the proportion of students in the HFZ; by 2018–19, 66.0% of students with very poor, 68.9% with poor, 72.0% with wealthy, and 76.1% with very wealthy home neighborhood SES were in the HFZ for push-ups. For all stratified curl-up models, the *p*-values for test for trend and the *p*-values for relative difference in trends by sex, grade level, race/ethnicity, and home neighborhood SES were < 0.001.

## Discussion

In this study analyzing standardized population-level data from 2006/07–2018/19 for NYC 4th−12th grade youth, we saw an overall increase in performance in individual fitness tests for aerobic capacity, push-ups, and curl-ups across the 13-year period, with the greatest improvements seen for aerobic capacity. While aerobic capacity increased for students overall, within subgroup differences remained relatively stable, demonstrating minimal reduction of inequities by sex, grade type, or home neighborhood SES, and a slight increase in disparities by race/ethnicity between non-Hispanic White and both non-Hispanic Black and Hispanic students. These findings signify that continued, strengthened, targeted interventions are necessary to close sociodemographic fitness gaps. Further, while the large relative increase (44%) in aerobic capacity over the 13-year period is encouraging, by the study's end, only 36% of New York City 4th−12th grade students were meeting age- and sex-specific standards for aerobic capacity. This demonstrates substantial work is still needed to increase students' cardiorespiratory health.

No other known US-based studies have examined trends in student aerobic capacity and muscular strength and endurance over this same period, making comparisons to other US populations challenging. However, a systematic review of global physical fitness trends from 2006–19 reported declining trends in aerobic capacity and muscular strength and endurance for students ages 4–18 (13), suggesting that work happening in the NYCDOE across this time period to improve student fitness met some success.

There could be a few reasons why NYC saw increased student aerobic capacity over this time-period. During the last four study years (2015–16 to 2018–19), the NYCDOE invested significant funding into improving physical education (PE) across city schools through the PE Works program. Longitudinal examination of that program using causal methods demonstrates PE Works led to a four percentage-point increase in the proportion of elementary students in the HFZ for aerobic capacity, which likely accounts for the increase in elementary students' cardiorespiratory fitness performance during the last few years of the current study (28). However, even prior to PE Works, aerobic capacity was increasing citywide. One hypothesis is that this could be due, in part, to improvements in test preparation over time, including increased attention paid by teachers to train students throughout the school year to take the FITNESSGRAM, or to improved adherence to testing protocol. It could also be attributed, in small part, to consistent statistically significant declines in the prevalence of obesity seen over the same time period among NYC students (29), given the known association between obesity and lower cardiorespiratory fitness in this population (30).

The differences in fitness performance by sex and grade level, wherein boys and elementary/middle school students are outperforming girls and high school students, respectively, seen in the present study are consistent with national findings. Data from 2012–2015 from an urban Missouri school district showed that 58% of boys were in the HFZ for aerobic capacity, compared to only 30% of girls (31). Elementary school boys have demonstrated higher aerobic fitness, speed, strength, and push-up performance compared with elementary school girls (32–34). Longitudinal FITNESSGRAM data from Georgia from 2011–14, show increases in HFZ achievements for aerobic capacity were largest among elementary schoolers, with smaller changes among middle and high schoolers (35).

It is also possible data quality could be biasing differences seen in HFZ achievement by grade level, as FITNESSGRAM data among NYC high school students has been reported to be of general lower quality compared to that of Kindergarten−8th graders (22). Another plausible hypothesis is that as students age, poorer fitness test performance is not a true marker of low fitness, but rather an artifact of lack of effort and disinterest in completing maximal fitness tests, such as the PACER, which is both physically taxing and effort-dependent. Further exploration of the NYC high school FITNESSGRAM data is necessary.

These data also demonstrated a clear dose-response relationship with home neighborhood SES and the proportion of students in the HFZ for all three fitness tests, with students with very wealthy home neighborhood SES consistently performing the best and students with very poor home neighborhood SES performing the worst. Prior studies have also showed differences in student fitness by SES, with students having lower odds of meeting HFZ standards when attending a school serving a greater percentage of economically disadvantaged students (36, 37). For example, in a study of schools across the US participating in the NFL PLAY60 Partnership Project program in 2016, student SES was found to be a significant predictor of HFZ achievement for aerobic capacity (11).

Notably, while differences in the proportion of students meeting the HFZ for aerobic capacity remained relatively stable within subgroups by sex, grade level, and home neighborhood SES, gaps in achieving the HFZ for aerobic capacity between non-Hispanic White compared to Non-Hispanic Black and Hispanic students grew slightly (by 1.9 and 2.6 percentage-points, respectively). Disparities in youth fitness by race/ethnicity are well documented in studies across the U.S. (11). A 2011 study of Wisconsin adolescents found significant differences in cardiorespiratory fitness among non-Hispanic White students and students of color. A 2015 study of students in the Midwest also showed non-White students scored lower on tests for both aerobic capacity and muscular strength and endurance (38).

Racial and ethnic differences in youth fitness are primarily due to a combination of socioeconomic factors, environmental conditions, and systemic inequities, rather than inherent biological differences (39, 40), emphasizing the need for policies and programs that target the root causes of these inequities.

In combination, these findings highlight the need for continued, targeted, systems- and environmental-level work to address the persistent disparities in fitness performance by sex, grade level, and home neighborhood SES, as well as the widening disparities by race/ethnicity. Whole school efforts, such as the development and execution of Comprehensive School Physical Activity Plans (CSPAP), show promise for increasing student physical activity and cardiorespiratory fitness in low-income elementary schools (41, 42), especially when schools allocate a within-school leader to spearhead coordination and implementation efforts (43). Further, ensuring adequate staffing for PE (i.e., funding PE teachers) is critical to address existing disparities in student fitness performance (44, 45), as is ensuring PE classes are taught with cultural competency, humility, and inclusion at the forefront (46, 47). Finally, ensuring there are strong state-, district-, and school-level policies that govern, evaluate, and fund physical activity and PE, as well as a school culture that prioritizes learning and health, can help ensure the prioritization and implementation of fitness-enhancing strategies in low-resource school settings (48, 49).

### Strengths and limitations

This study provides the most recent estimates of HFZ prevalence for aerobic capacity and muscular strength and endurance among NYC public school youth ages 9–19. We capitalize on the robust NYC FITNESSGRAM system, which has collected data for over a decade using standardized training and protocols with optimized fidelity across measurements (18, 50, 51). We use data from nearly 7 million observations of over 1.6 million unique students from the nation's largest, and among the most socioeconomically and racially/ethnically diverse, school districts. Further, these analyses uniquely draw from individual-level longitudinal data that standardize fitness performance scores during shifts over time in measurement and reporting criteria. While other reports describe youth fitness across other settings that administer the FITNESSGRAM (52–54), they do not present data over time, they present only aggregate findings, do not account for changes in HFZ reporting standards, and combine fitness with body composition measures, despite strong evidence demonstrating that weight status is conceptually distinct from fitness (15).

This work is not without limitations. First, given that NYC FITNESSGRAM data are not collected for research purposes, there is potential for systematic bias and random and/or differential measurement error, including variation across school sites where school staff may differ in their testing protocol, despite formal training and protocols which are designed to maximize consistency across administers (i.e., manuals, video-based training, site-visits). Second, our analysis excluded NYC private, charter, and special education students, representing 19.4, 13, and 2% of K-12 students, respectively (55), which limits the generalizability of these findings to all NYC students. Further, the NYC^®^ begins cardiorespiratory and muscular strength and endurance data collection in the 4th grade, precluding our ability to describe fitness trends for the city's K-3rd grade population. Third, fitness data was missing for 22% of eligible student observations. However, to address this, the measured population was weighted to be representative of the enrolled population, accounting for both individual- and school-level characteristics. Fourth, despite overall increases in student fitness from 2006/07–2018/19, all data are observational, limiting our ability to establish causal relationships with any concurrent policy or programmatic intervention. Finally, these data are inclusive on NYC students only, thus limiting generalizability to students in different school districts with different sociodemographic compositions.

## Conclusion

In this longitudinal examination of standardized population-level fitness data across 13 school years for NYC 4th−12th grade youth, we saw an overall improvement in performance on all three tests, with the largest gains seen for student aerobic capacity. However, the data did not demonstrate a narrowing of differences in aerobic capacity performance by sex, grade type, or home neighborhood SES, and saw a slight widening of disparities in performance by race/ethnicity, with the gap in performance between non-Hispanic White and non-Hispanic Black and Hispanic students growing slightly. Continued longitudinal monitoring of student fitness is critical to identify and support additional policy and programmatic interventions to address the persistent disparities in NYC youth aerobic capacity and muscular strength and endurance by sex, grade level, race/ethnicity, and socioeconomic status.

## Data Availability

The data analyzed in this study is subject to the following licenses/restrictions: the authors do not have permission to share data. Requests to access these datasets should be directed to sday@health.nyc.gov.
