# Supplementary material for: Trends in 4th−12th grade students' aerobic capacity and muscular strength and endurance: New York City public school students, 2006–2019
Source: Front Public Health. 2026 Feb 18;14:1682913. doi: 10.3389/fpubh.2026.1682913 (PMC12957200; doi:10.3389/fpubh.2026.1682913)
Supplement: Supplementary file 1 [file Table_1.docx]

**Appendix Table 1: Sociodemographic characteristics of unweighted New York City public school student sample (n_unweighted_=1,631,523 students, n_unweighted_=6,748,265 observations), grades 4–12**

|  | 2006/  07 | 2007/  08 | 2008/  09 | 2009/  10 | 2010/  11 | 2011/  12 | 2012/  13 | 2013/  14 | 2014/  15 | 2015/  16 | 2016/  17 | 2017  /18 | 2018/  19 |
| --- | --- | --- | --- | --- | --- | --- | --- | --- | --- | --- | --- | --- | --- |
| All Students, % (n) | 100  (243,350) | 100  (387,904) | 100  (486,005) | 100  (533,370) | 100  (551,385) | 100  (561,832) | 100  (565,049) | 100  (574,204) | 100  (572,022) | 100  (570,188) | 100  (564,970) | 100  (571,628) | 100  (566,358) |
| Sex | | | | | | | | | | | | | |
| Female | 49.7 | 49.4 | 49.7 | 49.5 | 49.4 | 49.3 | 49.3 | 49.2 | 49.1 | 49.1 | 49.1 | 49.1 | 48.9 |
| Male | 50.3 | 50.6 | 50.3 | 50.5 | 50.6 | 50.7 | 50.7 | 50.8 | 50.9 | 50.9 | 50.9 | 50.9 | 51.1 |
| Grade Level | | | | | | | | | | | | | |
| Elementary (4^th^-5^th^ grades) | 34.9 | 26.4 | 24.3 | 24.0 | 24.2 | 23.8 | 23.3 | 23.1 | 23.2 | 23.8 | 24.1 | 23.8 | 23.6 |
| Middle (6^th^-8^th^ grades) | 46.5 | 39.3 | 37.0 | 36.3 | 35.7 | 35.3 | 34.6 | 34.1 | 33.7 | 33.3 | 33.3 | 33.2 | 33.9 |
| High (9^th^-12^th^ grades) | 18.6 | 34.3 | 38.7 | 39.7 | 40.1 | 40.8 | 42.1 | 42.8 | 43.1 | 43.0 | 42.6 | 42.9 | 42.5 |
| Race | | | | | | | | | | | | | |
| Asian and/or Pacific Islander | 18.2 | 17.5 | 16.4 | 16.5 | 16.7 | 17.0 | 17.2 | 17.6 | 17.8 | 18.2 | 18.8 | 18.9 | 19.1 |
| Non-Hispanic Black | 29.6 | 30.7 | 30.8 | 30.2 | 29.7 | 28.7 | 27.9 | 26.8 | 25.8 | 24.9 | 23.9 | 23.3 | 22.6 |
| Hispanic | 34.2 | 35.5 | 37.9 | 38.8 | 39.1 | 39.3 | 39.5 | 39.8 | 40.1 | 40.0 | 40.0 | 40.3 | 40.4 |
| Non-Hispanic white | 17.5 | 15.8 | 14.4 | 14.0 | 14.0 | 14.4 | 14.6 | 14.9 | 15.1 | 15.4 | 15.7 | 15.7 | 15.8 |
| Other^a^ | 0.5 | 0.5 | 0.5 | 0.5 | 0.6 | 0.7 | 0.8 | 1.0 | 1.2 | 1.4 | 1.6 | 1.8 | 2.0 |
| Primary language spoken at home | | | | | | | | | | | | | |
| English | 56.1 | 55.2 | 55.8 | 55.8 | 55.7 | 55.5 | 55.3 | 54.9 | 54.8 | 54.6 | 54.1 | 54.9 | 54.0 |
| Spanish | 22.9 | 24.3 | 25.3 | 25.4 | 25.3 | 25.2 | 25.2 | 25.2 | 25.2 | 24.9 | 24.9 | 24.8 | 24.8 |
| Other language | 21.0 | 20.5 | 18.9 | 18.7 | 18.9 | 19.3 | 19.5 | 19.9 | 20.0 | 20.5 | 21.0 | 21.2 | 21.3 |
| Place of birth | | | | | | | | | | | | | |
| US | 80.2 | 78.6 | 79.3 | 79.6 | 79.6 | 79.9 | 80.2 | 80.3 | 80.5 | 80.6 | 80.2 | 79.9 | 79.9 |
| Foreign | 19.8 | 21.4 | 20.7 | 20.4 | 20.4 | 20.1 | 19.8 | 19.7 | 19.5 | 19.4 | 19.8 | 20.1 | 20.1 |
| Household poverty^b^ | | | | | | | | | | | | | |
| Low | 20.0 | 24.7 | 25.5 | 15.8 | 15.6 | 17.8 | 26.4 | 25.7 | 26.9 | 28.3 | 28.5 | 24.6 | 26.1 |
| High | 80.0 | 75.3 | 74.5 | 84.2 | 84.4 | 82.2 | 73.6 | 74.3 | 73.1 | 71.7 | 71.5 | 75.4 | 73.9 |
| Home neighborhood SES^c^ | | | | | | | | | | | | | |
| Very wealthy (0% to <10%) | 22.7 | 21.9 | 20.3 | 19.7 | 19.6 | 19.8 | 20.0 | 20.3 | 20.5 | 20.9 | 21.3 | 21.5 | 21.8 |
| Wealthy (10% to <20%) | 30.2 | 30.0 | 28.4 | 27.8 | 27.8 | 27.9 | 27.7 | 27.9 | 27.9 | 28.1 | 28.4 | 28.6 | 28.7 |
| Poor (20% to <30%) | 23.2 | 23.6 | 24.1 | 24.2 | 24.3 | 24.4 | 24.2 | 24.2 | 24.1 | 24.1 | 24.0 | 23.8 | 23.7 |
| Very poor (30% to 100%) | 23.9 | 24.6 | 27.2 | 28.2 | 28.2 | 27.9 | 28.0 | 27.7 | 27.4 | 26.9 | 26.3 | 26.2 | 25.8 |

^a^ Students not reporting Hispanic, non-Hispanic Black, non-Hispanic White, or Asian/Pacific Islander race/ethnicity in a school year are classified as “other,” which includes those reporting multiple races, parent refusal, or missing data. While a distinct racial classification, American Indian/Native Alaskan students are also grouped as “other” due to the small sample size.

^b^ Individual student household poverty (high vs. low) was based on student eligibility/non-eligibility for free/reduced price school meals through the National School Lunch Program which provides meal assistance according to household income at or below 185% of the federal poverty level.

^c^ Neighborhood socioeconomic status was defined according to American Community Survey 2008-2012 data as the percentage of households in the students’ home census tract living below the federal poverty threshold and defined according to the Census 2010 boundaries.
