# Supplementary material for: Trends in 4th−12th grade students' aerobic capacity and muscular strength and endurance: New York City public school students, 2006–2019
Source: Front Public Health. 2026 Feb 18;14:1682913. doi: 10.3389/fpubh.2026.1682913 (PMC12957200; doi:10.3389/fpubh.2026.1682913)
Supplement: Supplementary file 2 [file Table_2.docx]

**Appendix Table 2: Adjusted proportion^a^ of students meeting Healthy Fitness Zone^b^ standards for cardiorespiratory fitness and muscular strength and endurance for New York City public school students grades 4-12 (n_weighted_=8,523,877 observations), 2006/7-2018/19, by student sex**

|  | 2006/  07  % ± SD | 2007/  08  % ± SD | 2008/  09  % ± SD | 2009/  10  % ± SD | 2010/  11  % ± SD | 2011/  12  % ± SD | 2012/  13  % ± SD | 2013/  14  % ± SD | 2014/  15  % ± SD | 2015/  16  % ± SD | 2016/  17  % ± SD | 2017  /18  % ± SD | 2018/  19  % ± SD | Relative change  2006/07 to 2018/19 | p-value for test for trend ^c^ | p-value for relative difference in trend, by sex^d^ |
| --- | --- | --- | --- | --- | --- | --- | --- | --- | --- | --- | --- | --- | --- | --- | --- | --- |
| **Aerobic Capacity** | | | | | | | | | | | | | | | | |
| Female | 19.4 ± 0.91 | 18.8 ± 0.91 | 19.8 ± 0.72 | 20.5 ± 0.72 | 21.0 ± 0.73 | 22.2 ± 0.79 | 24.1 ± 0.64 | 24.1 ± 0.64 | 25.3 ± 0.62 | 27.4 ± 0.65 | 28.5 ± 0.63 | 29.7 ± 0.65 | 30.2 ± 0.64 | 60.6% | <0.001 | <0.001 |
| Male | 30.0 ± 1.21 | 28.6 ± 0.77 | 30.8 ± 0.75 | 32.8 ± 0.75 | 33.6 ± 0.74 | 35.0 ± 0.72 | 36.2 ± 0.70 | 38.1 ± 0.68 | 38.9 ± 0.67 | 40.8 ± 0.68 | 41.0 ± 0.66 | 41.5 ± 0.62 | 40.8 ± 0.64 | 42.7% | <0.001 | Ref |
| **Push-up** | | | | | | | | | | | | | | | | |
| Female | 52.0 ± 1.32 | 51.5 ± 0.84 | 51.4 ± 0.75 | 50.8 ± 0.77 | 50.9 ± 0.73 | 52.3 ± 0.73 | 53.5 ± 0.69 | 54.7 ± 0.71 | 56.9 ± 0.67 | 58.0 ± 0.69 | 57.9 ± 0.67 | 56.8 ± 0.68 | 56.4 ± 0.68 | 9.5% | <0.001 | <0.001 |
| Male | 58.6 ± 1.37 | 60.8 ± 0.69 | 60.2 ± 0.59 | 60.6 ± 0.61 | 61.0 ± 0.60 | 61.5 ± 0.57 | 61.5 ± 0.55 | 61.4 ± 0.54 | 61.3 ± 0.54 | 61.0 ± 0.54 | 59.7 ± 0.52 | 57.6 ± 0.54 | 55.8 ± 0.54 | -8.2% | <0.001 | Ref |
| **Curl-up** | | | | | | | | | | | | | | | | |
| Female | 63.9 ± 1.51 | 63.5 ± 0.98 | 62.9 ± 0.85 | 63.8 ± 0.81 | 65.3 ± 0.76 | 66.4 ± 0.71 | 67.2 ± 0.69 | 68.6 ± 0.65 | 70.5 ± 0.61 | 70.7 ± 0.60 | 69.9 ± 0.61 | 69.7 ± 0.61 | 69.4 ± 0.60 | 9.3% | <0.001 | <0.001 |
| Male | 66.1 ± 1.75 | 67.5 ± 0.93 | 68.1 ± 0.72 | 69.3 ± 0.68 | 70.8 ± 0.64 | 71.5 ± 0.61 | 72.2 ± 0.61 | 73.5 ± 0.58 | 74.1 ± 0.54 | 73.7 ± 0.56 | 72.8 ± 0.55 | 72.0 ± 0.55 | 71.6 ± 0.55 | 6.1% | <0.001 | Ref |

^a^ Estimated school year proportions derived from generalized estimating equation logistic models adjusted for student sex, age, race/ethnicity, place of birth, primary language spoken at home, and home neighborhood poverty level, with a random effect for school

^b^ Based on whether the student met the performance criteria for the Cooper Institute’s most recent sex- and age-specific Healthy Fitness Zones for each test

^c^ P-values for tests for trends over school years derived from logistic mixed effects models with a linear term for trend, adjusted for age, race/ethnicity, place of birth, primary language spoken at home, and home neighborhood poverty level with random effects for student and school.

^d^ P-values for relative differences in tests for trends between male and female students derived from logistic mixed effects models with a time*sex interaction term, adjusted for age, race/ethnicity, place of birth, primary language spoken at home, and home neighborhood poverty level with random effects for student and school.
