# Supplementary material for: Trends in 4th−12th grade students' aerobic capacity and muscular strength and endurance: New York City public school students, 2006–2019
Source: Front Public Health. 2026 Feb 18;14:1682913. doi: 10.3389/fpubh.2026.1682913 (PMC12957200; doi:10.3389/fpubh.2026.1682913)
Supplement: Supplementary file 3 [file Table_3.docx]

**Appendix Table 3: Adjusted proportion^a^ of students meeting Healthy Fitness Zone^b^ standards for cardiorespiratory fitness and muscular strength and endurance for New York City public school students grades 4-12 (n_weighted_=8,523,877 observations), 2006/7-2018/19, by grade level^c^**

|  | 2006/  07  % ± SD | 2007/  08  % ± SD | 2008/  09  % ± SD | 2009/  10  % ± SD | 2010/  11  % ± SD | 2011/  12  % ± SD | 2012/  13  % ± SD | 2013/  14  % ± SD | 2014/  15  % ± SD | 2015/  16  % ± SD | 2016/  17  % ± SD | 2017  /18  % ± SD | 2018/  19  % ± SD | Relative change  2006/07 to 2018/19 | p-value for test for trend ^d^ | p-value for relative difference in trend, by sex^e^ |
| --- | --- | --- | --- | --- | --- | --- | --- | --- | --- | --- | --- | --- | --- | --- | --- | --- |
| **Aerobic Capacity** | | | | | | | | | | | | | | | | |
| Elementary | 34.3 ± 1.25 | 33.6 ± 1.10 | 33.3 ± 1.03 | 34.4 ± 0.94 | 35.4 ± 0.96 | 37.3 ± 0.97 | 37.7 ± 0.97 | 38.8 ± 0.96 | 40.3 ± 0.94 | 42.7 ± 0.98 | 43.7 ± 1.00 | 45.3 ± 0.98 | 46.5 ± 0.96 | 35.6% | <0.001 | Ref |
| Middle | 27.6 ± 1.43 | 29.4 ± 1.29 | 31.4 ± 1.20 | 33.6 ± 1.21 | 34.2 ± 1.16 | 36.2 ± 1.20 | 37.2 ± 1.21 | 39.0 ± 1.26 | 39.6 ± 1.21 | 42.4 ± 1.27 | 42.8 ± 1.24 | 42.4 ± 1.21 | 42.5 ± 1.22 | 54.0% | <0.001 | <0.001 |
| High | 18.3 ± 1.75 | 15.6 ± 1.28 | 17.7 ± 1.07 | 18.6 ± 1.08 | 19.0 ± 1.10 | 19.5 ± 1.11 | 20.4 ± 1.05 | 22.2 ± 0.83 | 23.2 ± 0.83 | 24.3 ± 0.83 | 24.8 ± 0.81 | 26.1 ± 0.81 | 25.2 ± 0.83 | 37.7% | <0.001 | <0.001 |
| **Push-up** | | | | | | | | | | | | | | | | |
| Elementary | 58.2 ± 1.04 | 56.3 ± 0.97 | 57.3 ± 0.95 | 57.2 ± 0.95 | 56.9 ± 0.96 | 57.1 ± 0.94 | 57.2 ± 0.91 | 57.0 ± 0.91 | 57.8 ± 0.91 | 58.9 ± 0.91 | 58.3 ± 0.95 | 56.0 ± 0.93 | 56.1 ± 0.91 | -3.6% | <0.001 | Ref |
| Middle | 52.4 ± 1.17 | 53.3 ± 0.99 | 54.2 ± 0.97 | 55.2 ± 0.99 | 55.8 ± 0.96 | 57.2 ± 0.96 | 57.7 ± 0.93 | 58.1 ± 0.95 | 59.6 ± 0.92 | 60.2 ± 0.94 | 58.9 ± 0.91 | 56.9 ± 0.93 | 55.9 ± 0.94 | 6.7% | <0.001 | <0.001 |
| High | 56.0 ± 2.23 | 58.1 ± 1.13 | 56.3 ± 0.92 | 55.6 ± 1.00 | 55.8 ± 0.92 | 56.8 ± 0.91 | 57.6 ± 0.87 | 58.7 ± 0.86 | 59.4 ± 0.84 | 59.4 ± 0.86 | 59.0 ± 0.81 | 57.9 ± 0.84 | 56.2 ± 0.87 | 0.4% | <0.001 | 0.007 |
| **Curl-up** | | | | | | | | | | | | | | | | |
| Elementary | 63.9 ± 1.26 | 63.0 ± 1.19 | 64.5 ± 1.08 | 63.5 ± 1.12 | 64.7 ± 1.08 | 64.3 ± 1.06 | 63.6 ± 1.06 | 64.4 ± 1.06 | 65.4 ± 1.02 | 65.6 ± 0.99 | 64.8 ± 1.03 | 65.4 ± 0.99 | 66.8 ± 0.91 | 4.5% | <0.001 | Ref |
| Middle | 62.0 ± 1.62 | 62.1 ± 1.23 | 63.5 ± 1.10 | 65.8 ± 1.09 | 66.8 ± 1.08 | 67.8 ± 1.05 | 70.2 ± 0.96 | 70.9 ± 0.95 | 72.6 ± 0.92 | 73.2 ± 0.96 | 71.5 ± 0.99 | 70.8 ± 0.99 | 69.7 ± 0.98 | 12.4% | <0.001 | <0.001 |
| High | 67.7 ± 2.84 | 68.8 ± 1.49 | 67.4 ± 1.22 | 68.5 ± 1.12 | 70.7 ± 0.99 | 72.0 ± 0.89 | 72.3 ± 0.91 | 74.5 ± 0.82 | 75.4 ± 0.76 | 74.8 ± 0.77 | 74.7 ± 0.75 | 73.7 ± 0.74 | 72.9 ± 0.77 | 7.7% | <0.001 | <0.001 |

^a^ Estimated school year proportions derived from generalized estimating equation logistic models adjusted for student sex, age, race/ethnicity, place of birth, primary language spoken at home, and home neighborhood poverty level, with a random effect for school

^b^ Based on whether the student met the performance criteria for the Cooper Institute’s most recent sex- and age-specific Healthy Fitness Zones for each test

^c^ Grade level defined as elementary (grades 4-5), middle (grades 6-8), and high (grades 9-12)

^d^ P-values for tests for trends over school years derived from logistic mixed effects models with a linear term for trend, adjusted for age, race/ethnicity, place of birth, primary language spoken at home, and home neighborhood poverty level with random effects for student and school.

^e^ P-values for relative differences in tests for trends between male and female students derived from logistic mixed effects models with a time*grade level interaction term, adjusted for age, race/ethnicity, place of birth, primary language spoken at home, and home neighborhood poverty level with random effects for student and school.
