# Supplementary material for: Trends in 4th−12th grade students' aerobic capacity and muscular strength and endurance: New York City public school students, 2006–2019
Source: Front Public Health. 2026 Feb 18;14:1682913. doi: 10.3389/fpubh.2026.1682913 (PMC12957200; doi:10.3389/fpubh.2026.1682913)
Supplement: Supplementary file 4 [file Table_4.docx]

**Appendix Table 4: Adjusted proportion^a^ of students meeting Healthy Fitness Zone^b^ standards for cardiorespiratory fitness and muscular strength and endurance for New York City public school students grades 4-12 (n_weighted_=8,523,877 observations), 2006/7-2018/19, by student race/ethnicity^c^**

|  | 2006/  07  % ± SD | 2007/  08  % ± SD | 2008/  09  % ± SD | 2009/  10  % ± SD | 2010/  11  % ± SD | 2011/  12  % ± SD | 2012/  13  % ± SD | 2013/  14  % ± SD | 2014/  15  % ± SD | 2015/  16  % ± SD | 2016/  17  % ± SD | 2017  /18  % ± SD | 2018/  19  % ± SD | Relative change  2006/07 to 2018/19 | p-value for test for trend ^d^ | p-value for relative difference in trend, by sex^e^ |
| --- | --- | --- | --- | --- | --- | --- | --- | --- | --- | --- | --- | --- | --- | --- | --- | --- |
| **Aerobic Capacity** | | | | | | | | | | | | | | | | |
| Asian/PI | 25.2 ± 1.38 | 25.2 ± 1.17 | 27.7 ± 1.19 | 29.2 ± 1.23 | 29.9 ± 1.27 | 31.0 ± 1.21 | 31.4 ± 1.15 | 33.2 ± 1.11 | 33.0 ± 1.12 | 34.6 ± 1.07 | 35.7 ± 1.07 | 36.5 ± 1.02 | 36.8 ± 1.06 | 46.0% | <0.001 | <0.001 |
| NH-Black | 22.6 ± 1.24 | 21.5 ± 0.79 | 23.3 ± 0.78 | 24.6 ± 0.78 | 25.0 ± 0.75 | 26.6 ± 0.80 | 27.2 ± 0.75 | 29.0 ± 0.72 | 30.5 ± 0.72 | 32.1 ± 0.73 | 32.6 ± 0.71 | 33.0 ± 0.71 | 32.8 ± 0.68 | 45.1% | <0.001 | <0.001 |
| Hispanic | 23.9 ± 1.15 | 22.7 ± 0.90 | 23.8 ± 0.69 | 25.2 ± 0.66 | 25.8 ± 0.63 | 26.9 ± 0.64 | 27.8 ± 0.64 | 29.2 ± 0.63 | 30.4 ± 0.60 | 32.7 ± 0.64 | 32.8 ± 0.61 | 33.8 ± 0.61 | 33.4 ± 0.62 | 39.7% | <0.001 | <0.001 |
| NH-White | 31.6 ± 1.61 | 30.8 ± 1.78 | 32.2 ± 1.57 | 32.9 ± 1.63 | 34.3 ± 1.63 | 35.6 ± 1.68 | 37.1 ± 1.62 | 38.7 ± 1.35 | 39.4 ± 1.28 | 41.5 ± 1.33 | 42.9 ± 1.23 | 43.6 ± 1.15 | 43.7 ± 1.19 | 38.3% | <0.001 | Ref |
| **Push-up** | | | | | | | | | | | | | | | | |
| Asian/PI | 58.9 ± 1.60 | 59.0 ± 1.10 | 59.7 ± 1.07 | 60.3 ± 1.21 | 60.5 ± 1.08 | 61.9 ± 1.08 | 62.4 ± 1.07 | 62.4 ± 1.11 | 63.2 ± 1.07 | 63.4 ± 1.06 | 63.1 ± 0.99 | 61.0 ± 0.98 | 60.5 ± 1.03 | 2.7% | <0.001 | <0.001 |
| NH-Black | 55.0 ± 1.54 | 56.7 ± 0.89 | 55.8 ± 0.70 | 55.4 ± 0.72 | 55.5 ± 0.70 | 55.7 ± 0.73 | 56.3 ± 0.69 | 57.3 ± 0.68 | 58.7 ± 0.67 | 58.8 ± 0.69 | 57.4 ± 0.65 | 56.3 ± 0.66 | 55.1 ± 0.65 | 0.2% | <0.001 | <0.001 |
| Hispanic | 51.5 ± 1.29 | 52.1 ± 0.79 | 51.5 ± 0.69 | 51.4 ± 0.66 | 51.4 ± 0.66 | 52.2 ± 0.64 | 52.7 ± 0.60 | 52.7 ± 0.60 | 53.7 ± 0.61 | 54.0 ± 0.62 | 53.0 ± 0.62 | 51.2 ± 0.63 | 49.7 ± 0.64 | -3.5% | <0.001 | <0.001 |
| NH-White | 63.3 ± 1.43 | 63.6 ± 0.95 | 64.3 ± 1.00 | 64.5 ± 1.23 | 65.5 ± 1.09 | 67.5 ± 0.98 | 68.0 ± 0.97 | 69.4 ± 0.95 | 70.0 ± 0.87 | 71.1 ± 0.86 | 71.0 ± 0.83 | 69.5 ± 0.86 | 68.8 ± 0.84 | 8.7% | <0.001 | Ref |
| **Curl-up** | | | | | | | | | | | | | | | | |
| Asian/PI | 71.2 ± 2.15 | 70.9 ± 1.41 | 71.2 ± 1.27 | 72.0 ± 1.28 | 73.4 ± 1.13 | 74.5 ± 1.05 | 74.9 ± 1.02 | 76.1 ± 0.98 | 77.1 ± 0.95 | 76.5 ± 0.94 | 75.9 ± 0.95 | 75.2 ± 0.93 | 75.4 ± 0.96 | 5.9% | <0.001 | <0.001 |
| NH-Black | 62.9 ± 1.75 | 63.9 ± 1.08 | 63.8 ± 0.86 | 64.6 ± 0.87 | 66.3 ± 0.82 | 67.2 ± 0.80 | 67.9 ± 0.74 | 69.3 ± 0.69 | 70.6 ± 0.65 | 70.6 ± 0.69 | 69.8 ± 0.68 | 69.1 ± 0.66 | 68.9 ± 0.66 | 9.5% | <0.001 | <0.001 |
| Hispanic | 61.6 ± 1.76 | 62.3 ± 1.04 | 62.1 ± 0.86 | 63.5 ± 0.77 | 65.1 ± 0.74 | 65.5 ± 0.71 | 66.3 ± 0.69 | 67.7 ± 0.66 | 68.9 ± 0.61 | 68.7 ± 0.61 | 67.5 ± 0.62 | 67.0 ± 0.62 | 66.2 ± 0.61 | 7.5% | <0.001 | <0.001 |
| NH-White | 73.1 ± 1.79 | 72.7 ± 1.24 | 73.6 ± 1.23 | 73.7 ± 1.33 | 74.9 ± 1.13 | 76.4 ± 1.03 | 76.9 ± 1.03 | 77.9 ± 0.96 | 79.1 ± 0.86 | 79.1 ± 0.82 | 78.5 ± 0.85 | 78.3 ± 0.81 | 78.3 ± 0.83 | 7.1% | <0.001 | Ref |

^a^ Estimated school year proportions derived from generalized estimating equation logistic models adjusted for student sex, age, race/ethnicity, place of birth, primary language spoken at home, and home neighborhood poverty level, with a random effect for school

^b^ Based on whether the student met the performance criteria for the Cooper Institute’s most recent sex- and age-specific Healthy Fitness Zones for each test

^c^ Categories are Asian/Pacific Islander, Non-Hispanic Black, Hispanic/Latino, and Non-Hispanic White. Students not reporting Hispanic, non-Hispanic Black, non-Hispanic White, or Asian/Pacific Islander race/ethnicity in a school year are classified as “other,” which includes those reporting multiple races, parent refusal, or missing data. While a distinct racial classification, American Indian/Native Alaskan students are also grouped as “other” due to the small sample size. Although the classification of the “other” race/ethnicity category is not coherent, we use it for modeling and analysis with the aim to reduce its use. In consideration of these limitations, prevalence estimates for students classified as “other” race/ethnicity are not provided.

^d^ P-values for tests for trends over school years derived from logistic mixed effects models with a linear term for trend, adjusted for age, race/ethnicity, place of birth, primary language spoken at home, and home neighborhood poverty level with random effects for student and school.

^e^ P-values for relative differences in tests for trends between male and female students derived from logistic mixed effects models with a time*race/ethnicity interaction term, adjusted for age, race/ethnicity, place of birth, primary language spoken at home, and home neighborhood poverty level with random effects for student and school.
