# Supplementary material for: Trends in 4th−12th grade students' aerobic capacity and muscular strength and endurance: New York City public school students, 2006–2019
Source: Front Public Health. 2026 Feb 18;14:1682913. doi: 10.3389/fpubh.2026.1682913 (PMC12957200; doi:10.3389/fpubh.2026.1682913)
Supplement: Supplementary file 5 [file Table_5.docx]

**Appendix Table 5: Adjusted proportion^a^ of students meeting Healthy Fitness Zone^b^ standards for cardiorespiratory fitness and muscular strength and endurance for New York City public school students grades 4-12 (n_weighted_=8,523,877 observations), 2006/7-2018/19, by student home neighborhood SES^c^**

|  | 2006/  07  % ± SD | 2007/  08  % ± SD | 2008/  09  % ± SD | 2009/  10  % ± SD | 2010/  11  % ± SD | 2011/  12  % ± SD | 2012/  13  % ± SD | 2013/  14  % ± SD | 2014/  15  % ± SD | 2015/  16  % ± SD | 2016/  17  % ± SD | 2017  /18  % ± SD | 2018/  19  % ± SD | Relative change  2006/07 to 2018/19 | p-value for test for trend ^d^ | p-value for relative difference in trend, by sex^e^ |
| --- | --- | --- | --- | --- | --- | --- | --- | --- | --- | --- | --- | --- | --- | --- | --- | --- |
| **Aerobic Capacity** | | | | | | | | | | | | | | | | |
| Very wealthy | 29.4 ± 1.33 | 28.6 ± 1.35 | 29.9 ± 1.28 | 31.0 ± 1.27 | 32.1 ± 1.29 | 33.7 ± 1.31 | 34.7 ± 1.24 | 36.6 ± 1.07 | 37.5 ± 1.05 | 39.0 ± 1.09 | 40.6 ± 1.00 | 41.3 ± 0.94 | 41.1 ± 0.96 | 39.8% | <0.001 | Ref |
| Wealthy | 24.4 ± 1.14 | 24.5 ± 0.89 | 26.4 ± 0.85 | 28.0 ± 0.88 | 28.5 ± 0.89 | 29.9 ± 0.89 | 30.1 ± 0.85 | 31.3 ± 0.81 | 32.2 ± 0.79 | 34.0 ± 0.80 | 34.6 ± 0.79 | 35.1 ± 0.77 | 34.7 ± 0.77 | 42.2% | <0.001 | <0.001 |
| Poor | 23.2 ± 1.04 | 21.8 ± 0.77 | 24.4 ± 0.72 | 25.9 ± 0.71 | 26.3 ± 0.70 | 27.6 ± 0.73 | 28.4 ± 0.72 | 30.2 ± 0.69 | 31.0 ± 0.66 | 33.2 ± 0.72 | 33.3 ± 0.70 | 33.9 ± 0.67 | 34.2 ± 0.69 | 47.4% | <0.001 | <0.001 |
| Very poor | 23.1 ± 1.31 | 21.5 ± 0.94 | 22.3 ± 0.71 | 23.3 ± 0.65 | 24.3 ± 0.64 | 25.2 ± 0.68 | 26.5 ± 0.69 | 28.4 ± 0.69 | 29.6 ± 0.67 | 31.9 ± 0.70 | 32.1 ± 0.70 | 33.6 ± 0.72 | 33.4 ± 0.73 | 44.6% | <0.001 | <0.001 |
| **Push-up** | | | | | | | | | | | | | | | | |
| Very wealthy | 61.8 ± 1.44 | 62.4 ± 0.86 | 62.5 ± 0.92 | 63.1 ± 1.01 | 63.9 ± 0.94 | 65.2 ± 0.89 | 66.0 ± 0.86 | 67.3 ± 0.85 | 68.2 ± 0.82 | 68.1 ± 0.83 | 67.7 ± 0.79 | 65.9 ± 0.80 | 64.9 ± 0.78 | 5.0% | <0.001 | Ref |
| Wealthy | 56.7 ± 1.42 | 57.8 ± 0.82 | 57.5 ± 0.76 | 57.8 ± 0.81 | 58.0 ± 0.76 | 59.2 ± 0.74 | 59.7 ± 0.72 | 60.1 ± 0.74 | 61.0 ± 0.74 | 61.1 ± 0.74 | 60.6 ± 0.69 | 58.2 ± 0.73 | 57.2 ± 0.73 | 0.9% | <0.001 | <0.001 |
| Poor | 53.3 ± 1.30 | 54.3 ± 0.79 | 54.2 ± 0.67 | 53.5 ± 0.70 | 53.6 ± 0.68 | 54.6 ± 0.69 | 55.1 ± 0.66 | 55.2 ± 0.69 | 56.4 ± 0.69 | 56.9 ± 0.69 | 55.9 ± 0.69 | 54.3 ± 0.68 | 53.2 ± 0.67 | -0.2% | <0.001 | <0.001 |
| Very poor | 51.2 ± 1.48 | 52.2 ± 0.84 | 51.4 ± 0.69 | 51.1 ± 0.68 | 51.1 ± 0.69 | 51.3 ± 0.70 | 51.8 ± 0.65 | 52.2 ± 0.64 | 53.3 ± 0.63 | 53.9 ± 0.64 | 52.8 ± 0.64 | 51.8 ± 0.64 | 50.6 ± 0.67 | -1.2% | 0.0009 | <0.001 |
| **Curl-up** | | | | | | | | | | | | | | | | |
| Very wealthy | 72.1 ± 1.51 | 72.0 ± 1.06 | 72.2 ± 0.98 | 72.3 ± 1.10 | 74.0 ± 0.97 | 75.2 ± 0.91 | 75.6 ± 0.88 | 76.7 ± 0.86 | 77.9 ± 0.76 | 77.5 ± 0.76 | 76.5 ± 0.77 | 76.1 ± 0.72 | 76.1 ± 0.74 | 5.5% | <0.001 | Ref |
| Wealthy | 67.5 ± 1.69 | 67.7 ± 1.02 | 68.1 ± 0.92 | 69.2 ± 0.90 | 70.6 ± 0.82 | 71.7 ± 0.76 | 72.4 ± 0.73 | 73.5 ± 0.71 | 74.3 ± 0.67 | 74.2 ± 0.69 | 73.4 ± 0.68 | 72.5 ± 0.69 | 72.0 ± 0.70 | 6.7% | <0.001 | <0.001 |
| Poor | 63.1 ± 1.70 | 63.5 ± 0.97 | 63.8 ± 0.82 | 64.9 ± 0.79 | 66.6 ± 0.75 | 67.2 ± 0.73 | 68.0 ± 0.75 | 69.5 ± 0.71 | 70.9 ± 0.68 | 70.6 ± 0.68 | 69.9 ± 0.71 | 69.2 ± 0.68 | 68.9 ± 0.64 | 9.2% | <0.001 | <0.001 |
| Very poor | 59.3 ± 1.93 | 60.8 ± 1.10 | 60.4 ± 0.90 | 61.8 ± 0.76 | 63.3 ± 0.78 | 63.9 ± 0.77 | 64.7 ± 0.74 | 66.3 ± 0.71 | 67.6 ± 0.69 | 67.8 ± 0.70 | 66.7 ± 0.69 | 66.6 ± 0.68 | 66.0 ± 0.70 | 11.3% | <0.001 | <0.001 |

^a^ Estimated school year proportions derived from generalized estimating equation logistic models adjusted for student sex, age, race/ethnicity, place of birth, primary language spoken at home, and home neighborhood poverty level, with a random effect for school

^b^ Based on whether the student met the performance criteria for the Cooper Institute’s most recent sex- and age-specific Healthy Fitness Zones for each test

^c^ Home neighborhood socioeconomic status (SES) was defined according to American Community Survey 2008-2012 data as the percentage of households in the students’ home census tract living below the federal poverty threshold and defined according to the Census 2010 boundaries.

^d^ P-values for tests for trends over school years derived from logistic mixed effects models with a linear term for trend, adjusted for age, race/ethnicity, place of birth, primary language spoken at home, and home neighborhood poverty level with random effects for student and school.

^e^ P-values for relative differences in tests for trends between male and female students derived from logistic mixed effects models with a time*home neighborhood SES interaction term, adjusted for age, race/ethnicity, place of birth, primary language spoken at home, and home neighborhood poverty level with random effects for student and school.
